# A Systematic Review of Methodology Used in Studies Aimed at Creating Charts of Fetal Brain Structures

**DOI:** 10.3390/diagnostics11060916

**Published:** 2021-05-21

**Authors:** Vera Donadono, Angelo Cavallaro, Nia W. Roberts, Christos Ioannou, Aris T. Papageorghiou, Raffaele Napolitano

**Affiliations:** 1 Fetal Medicine Unit, University College London Hospitals NHS Foundation Trust, London WC1E 6DB, UK; vera.donadono@nhs.net; 2 Nuffield Department of Women’s & Reproductive Health, John Radcliffe Hospital, University of Oxford, Oxford OX3 9DU, UK; angelo.cavallaro@ouh.nhs.uk (A.C.); christos.ioannou@ouh.nhs.uk (C.I.); aris.papageorghiou@wrh.ox.ac.uk (A.T.P.); 3 Bodleian Health Care Libraries, University of Oxford, Oxford OX3 9DU, UK; nia.roberts@bodleian.ox.ac.uk; 4 Oxford Maternal & Perinatal Health Institute, Green Templeton College, University of Oxford, Oxford OX3 9DU, UK; 5 Elizabeth Garrett Anderson Institute for Women′s Health, University College London, London WC1E 6DB, UK

**Keywords:** ultrasound, growth, parieto-occipital fissure, Sylvian fissure, anterior ventricle, posterior ventricle, transcerebellar diameter, cisterna magna

## Abstract

Ultrasound-based assessment of the fetal nervous system is routinely recommended at the time of the mid-trimester anatomy scan or at different gestations based on clinical indications. This review evaluates the methodological quality of studies aimed at creating charts for fetal brain structures obtained by ultrasound, as poor methodology could explain substantial variability in percentiles reported. Electronic databases (MEDLINE, EMBASE, Cochrane Library, and Web of Science) were searched from January 1970 to January 2021 to select studies on singleton fetuses, where the main aim was to construct charts on one or more clinically relevant structures obtained in the axial plane: parieto-occipital fissure, Sylvian fissure, anterior ventricle, posterior ventricle, transcerebellar diameter, and cisterna magna. Studies were scored against 29 predefined methodological quality criteria to identify the risk of bias. In total, 42 studies met the inclusion criteria, providing data for 45,626 fetuses. Substantial heterogeneity was identified in the methodological quality of included studies, and this may explain the high variability in centiles reported. In 80% of the studies, a high risk of bias was found in more than 50% of the domains scored. In conclusion, charts to be used in clinical practice and research should have an optimal study design in order to minimise the risk of bias and to allow comparison between different studies. We propose to use charts from studies with the highest methodological quality.

## 1. Introduction

Ultrasound-based assessment of the fetal nervous system is routinely recommended in most settings at the time of the mid-trimester fetal anatomy scan or at different gestations based on clinical indications [1,2]. This usually includes routine measurement of the lateral ventricle anteriorly (AV) and posteriorly (PV), the transcerebellar diameter (TCD), and the cisterna magna (CM). Additional measurements as part of an extended neurosonography examination have been proposed in order to assess gyration and sulcation disorders, such as the parieto-occipital fissure (POF) and the Sylvian fissure (SF). 

In previous systematic reviews of studies aimed at creating fetal and neonatal biometry charts, many studies were found to have high risks of bias. Such shortcomings of methodological design can become a source of substantial variability in percentiles reported, with differences in interpretation of the same measurement; ultimately, this can adversely influence clinical decision making [3,4]. Over the last five years, international prescriptive standards have been published in order to overcome the limitations inherent in such descriptive reference charts [5].

The objective of this systematic review was to evaluate the methodological quality of studies aimed to develop charts of fetal brain structures measured by ultrasound.

## 2. Materials and Methods

We conducted a systematic review of observational studies following the Preferred Reporting Item for Systematic Reviews and Meta-Analyses (PRISMA) IPD statement [6]. We searched the major electronic databases (MEDLINE, EMBASE, Cochrane Library, and Web of Science) and secondary reference sources from January 1970 to January 2021 to select studies on singleton fetuses aimed at creating charts on fetal brain structures growth.

Inclusion criteria for each study were (1) having as the main scope to construct charts on POF, SF, AV, PV, TCD, and CM; (2) published in English; (3) selection of normal singleton pregnancies; (4) acquisition of the image on routinely acquired transverse axial planes (transthalamic, transventricular, transcerebellar) [1]; and (5) growth charts developed beyond 14 weeks of gestation. No restriction for the ultrasound acquisition technique was applied (either from 2D pictures or images derived from 3D volumes and with transvaginal or transabdominal probe). Studies aiming at comparing different population groups or methods of imaging were excluded from the review.

The keyword search strategy was formulated in collaboration with a professional information specialist (NWR) and is presented in Table S1. Two reviewers (VD and RN) independently undertook a two-stage process to select the studies. In the first stage, they assessed abstracts and titles of all identified citations and selected potentially eligible studies. In the second stage, they obtained and assessed the full texts of the studies that fulfilled the inclusion criteria for evaluation. Disagreements regarding inclusion were resolved by consensus or by consultation with a third author (ATP). Reference lists of retrieved full-text articles were examined for additional, relevant citations.

Methodological quality criteria were defined a priori, using modified versions previously used to evaluate studies aimed at creating fetal growth charts and crown–rump length dating charts [3,4]. Table S2 reports the set of 29 quality criteria. Those criteria refer to three domains, namely, (1) study design (2) statistical methods, and (3) reporting methods.

All studies included were then scored against each criterion. The level of bias was defined as a dichotomous variable: 0 referred to a ‘high risk’, and 1 referred to a ‘low risk’. The overall risk score was defined by adding all scores across the whole set of criteria. Thus, the quality score for each item of the review could range from 0 (highest risk of bias) to 29 (lowest risk of bias). The assessment of the methodological quality was performed by two reviewers (RN and AC) for each study. Where disagreements arose, those were solved through consultation with a third reviewer (ATP).

### Statistical Analysis

Data from the review were coded and transferred to an Excel spreadsheet (Microsoft Corporation 2007, Redmond, WA, USA). The quality score (0–29) was reported in percentage dividing the actual score by 29 and multiplying per 100. The distribution of the 5th and the 95th centile in studies with a low and high risk of bias was evaluated.

We also evaluated the impact on centiles’ heterogeneity associated with poor study methodology in the most commonly measured brain structure—TCD.

## 3. Results

**Figure 1:**
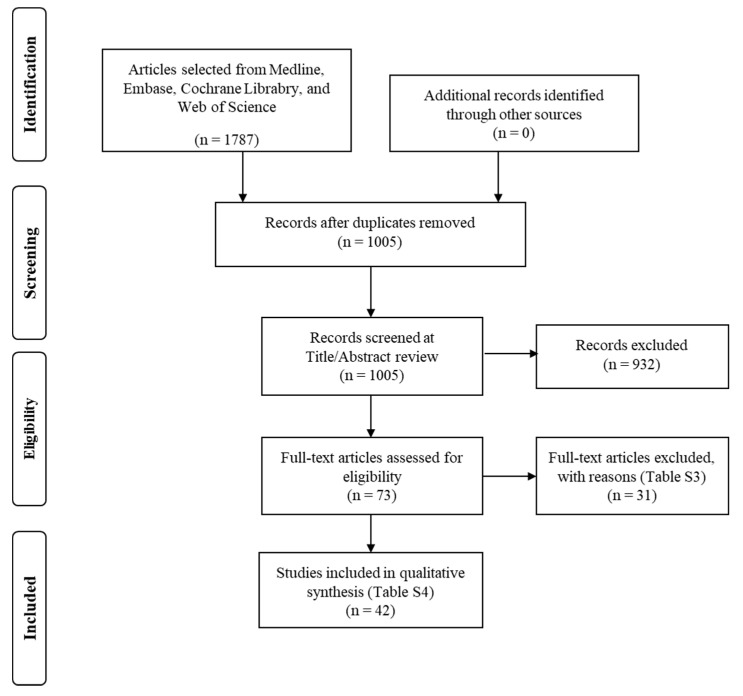
Flow diagram of the studies’ selection process.

From a total of 1005 records identified after database search, 73 were considered for potential inclusion (Figure 1). Excluded studies and reasons for exclusion are reported in Table S3. Finally, 42 studies, reported between January 1970 and 2021, met the inclusion criteria, and these provided data for 45,626 fetuses, included in the final analysis (Table 1) [7,8,9,10,11,12,13,14,15,16,17,18,19,20,21,22,23,24,25,26,27,28,29,30,31,32,33,34,35,36,37,38,39,40,41,42,43,44,45,46,47,48]. The median sample size of participating fetuses was 372.5 (range: 50 to 8313; 25th percentile: 175.8; 75th percentile: 709.3). Most studies created charts that covered a range of gestation; for example, if a study reported a chart from 20 to 40 weeks, this covered 21 weeks. The median of this coverage was 24 weeks (range: 7 to 30 weeks; 25th percentile: 18; 75th percentile: 27).

**Figure 2:**
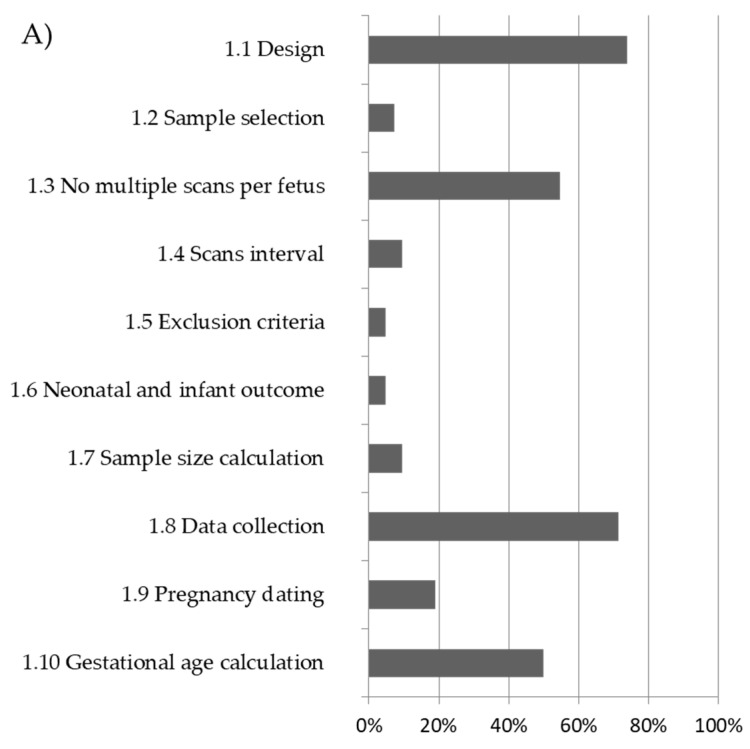

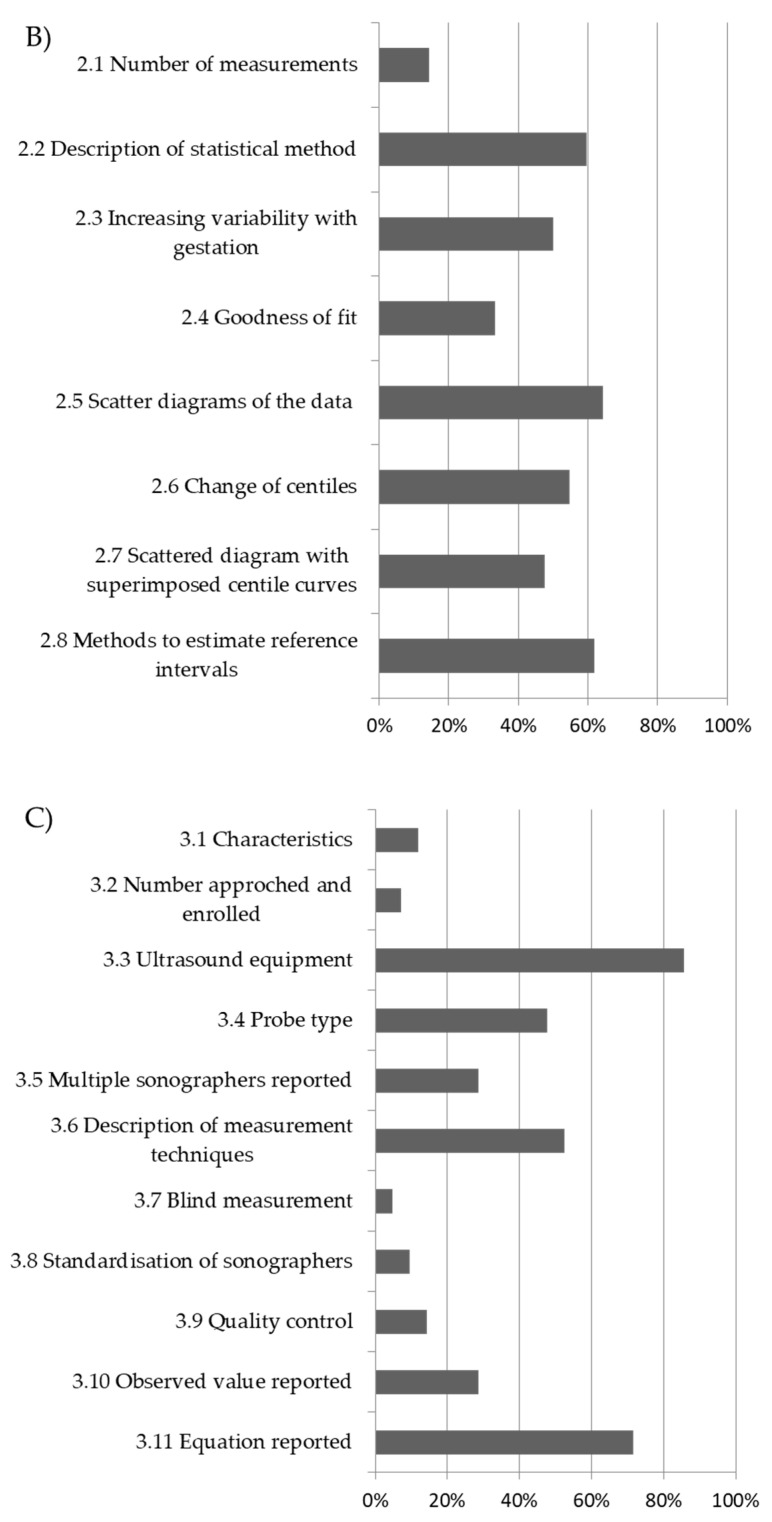
Overall methodological quality of included studies and percentage of low risk of bias: (**A**) study design; (**B**) statistical methods; (**C**) reporting methods.

Nine studies reported more than one fetal brain structure [9,10,17,32,34,36,41,42,43]. In addition, 4 studies reported charts for POF [9,10,17,36], 6 for SF [9,10,17,35,36,44], 3 for AV [22,36,43], 14 for PV [7,8,14,19,23,26,27,30,32,34,36,38,40,43], 20 for TCD [12,15,16,18,20,21,24,25,28,31,32,34,39,41,42,43,45,46,47,48], and 10 studies reported the CM [11,13,29,33,34,36,37,41,42,43]. There was substantial heterogeneity in the methodology used in studies aimed at creating charts for fetal brain structures. In 34 studies (80%), high risk of bias was found in >50% of the domains scored (Figure 2, Table S4).

The overall quality score in terms of low risk of bias ranged between 5 and 74%, 15 and 65%, and 5 and 86% for criteria analysed in the study design, statistical methods, and reporting methods domain, respectively (Figure 2). Overall, 30 studies (72%) undertook prospective data collection. Only 5 studies (12%) had a longitudinal design, and 26 studies (62%) had a cross-sectional design, whereas in all the remaining 11 studies (26%) the design was not reported. Only two studies (5%) had inclusion and exclusion criteria that were clearly stated and applied, and also reported detailed neonatal or infant outcomes (Table S4). Considering the domain of ‘statistical methods’, there was a low risk of bias in only 25 studies (60%); a regression equation was reported in order to calculate expected centiles in 30 studies (72%), and only in 14 studies (33%) was performed goodness of fit of the proposed model (Table S4, Figure 2). Most of the studies scored low on criteria concerning the reporting methods such as the description of the measurement technique (50%). Only six studies (14%) adopted a comprehensive strategy for ultrasound quality control—in four cases (9%) sonographers were standardised to the measuring technique, and in two (5%), cases measurements were taken in a blinded fashion.

**Figure 3:**
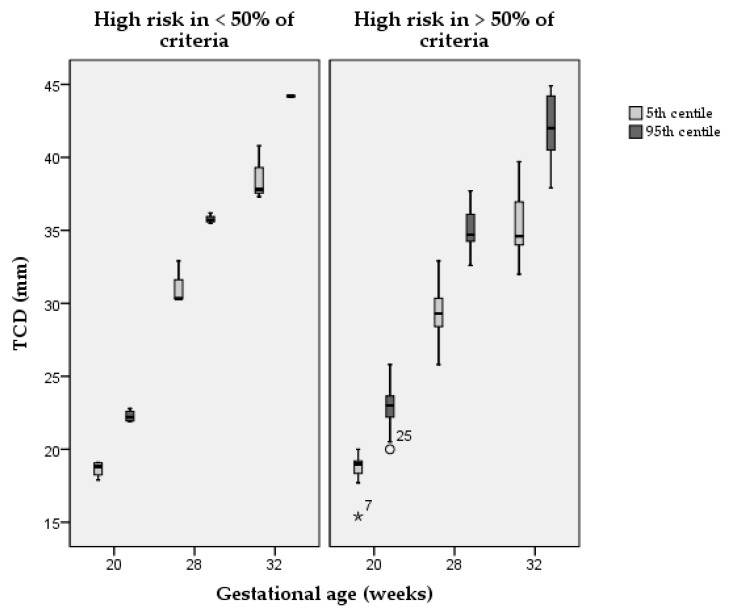
Distribution of the 5th and the 95th centile in studies with a high risk of bias in more or less than 50% of the quality criteria. TCD = transcerebellar diameter; ° = outlier; * = extreme outlier.

In Table 2, we present the centiles of each structure at three relevant gestational ages for those studies where this was possible—either reported by the authors or calculated when a relevant equation was reported. Figure 3 shows the distribution of the 5th and the 95th centile for the TCD in studies with a high risk of bias in more or less than 50% of the quality criteria. Studies with a lower risk of bias had a smaller distribution of centiles, compared with studies with a higher risk of bias at any of the three gestational ages analysed. The same analysis could not be reported for other structures in view of a low number of data points.

## 4. Discussion

The aim of this systematic review was to evaluate the methodology used in studies aimed at creating charts on specific fetal brain structures measured by ultrasound. Using a set of 29 predefined quality criteria on study design, statistical methods, and reporting methods, studies were scored as having a low or high risk of bias. This approach has been previously proposed in order to evaluate the quality of existing charts on fetal biometry and first-trimester dating [3,4].

In 34 out of 42 studies (80%), a high risk of bias was found in >50% of the domains scored (Figure 2). Only the studies by Napolitano et al. and Rodriguez-Sibaja et al. were at low risk of bias in a significantly high number of quality criteria, respectively, in 93% and 86%; all other studies were below 60% [36,39].

The highest potential for bias was noted in most of the criteria regarding the ‘study design’. Specifically, only two studies reported a low risk of bias in the definition of inclusion/exclusion criteria. In addition, only two studies described detailed neonatal and development outcomes [36,39]. In two other studies, there was neurological follow-up described, but this was never assessed with a standardised approach in all fetuses included. Thus, in the study by Hilpert et al. telephone follow-up was obtained at a minimum of 2 years of age in fetuses with PV measurement of 10 mm or more [26], and in the study by Farrell et al., most fetuses with PV measurements more than 8 mm had an unspecified follow-up that varied from 2 days to 12 months [19]. We believe that infant follow-up is essential if the aim is to create charts of brain structures. This is because pathological conditions may be prevalent, possibly affecting the resulting charts, and because many cases of developmental delay cannot currently be predicted by antenatal ultrasound. The proportion of infants with abnormal development in a study should confirm that they are representative of a healthy population.

The reason for this high source for bias identified in these fields is also probably related to the retrospective design (around 30% of the studies). Furthermore, in only four studies sample size estimation was performed [18,36,39,44], and only three studies had population-based sample selection [36,39,47], with all other studies reporting either convenience sampling or arbitrary recruitment or sampling methods that were not reported (Figure 2).

One area of significant bias in fetal biometry is associated with calliper placement not done in a blinded fashion [49] (considered in only two studies [36,39]). It is difficult to quantify the magnitude of this factor, but one might assume that for values close to cut-offs for referral or investigation (e.g., 10 mm for the PV); such lack of blinding may have a relevant impact on the resulting charts. The sonographer tends to over- or underestimate to generate referrals or avoid abnormality diagnosis, respectively. In addition, there was a lack of ultrasound quality control in 80% of the studies, which has been previously demonstrated to be useful in reducing measurement variability [50].

The high risk of bias in most of the criteria assessed may explain the substantial heterogeneity in the resulting centiles. For example, in the case of TCD at 32 weeks of gestation, the 50th centile according to Vinkesteijn et al. was equivalent to the 95th centile according to Hayata et al. and Hata et al. [24,25,48]. Likewise, the 95th centile according to Smith et al. was smaller than the 5th centile according to Serhatlioglu et al. (Table 2) [41,42]. In Figure 3, we show that the higher is the risk of bias score the higher is the variability in centiles reported of TCD for the three gestational age ranges considered. It is clear that such differences are not desirable since they can lead to false-positive or false-negative results. This also makes different studies difficult to compare in research.

Only nine studies reported the regression equation of the standard deviation—instead, they either did not report the equation or they just reported the mean standard deviation throughout gestation. This is an important limitation since a clear increase in variability with advancing gestation from visual assessment of scatter diagrams in those studies was observed. Hence, the changing standard deviation with advancing gestation should be taken into account and is needed for the accurate calculation of centiles.

### Strengths and Limitations of the Review

This is the first systematic review on the topic and it includes all currently published charts on POF, SF, AV, PV, TCD, and CM. A rigorous methodology based on predetermined quality criteria was applied and was based on previously published quality checklists used to evaluate studies on other aspects of fetal size [3,4]. We had no limitations on the year of inclusion of studies; it could be argued that, in a rapidly emerging field such as prenatal imaging, older studies should not be subjected to the same rigorous quality assessment as more recent ones. It is also fair to assume a gradual improvement in both the ultrasound technology and the statistical methods of data analysis over the decades. In fact, there was some evidence of improving study quality over time; the median quality score for the first half of the studies (between 1984 and 2008) was 9, while the median score for the latter half of the studies was 13. Nevertheless, the high risk of bias was time independent for many criteria such as inclusion/exclusion criteria, neonatal and infant outcome, sample selection, characteristics of the study population, measurement acquired blindly, and standardisation of the sonographers.

## 5. Conclusions

The use of a wide range of reference charts can affect both clinical assessment and research on the development of new technologies associated with antenatal ultrasound [51,52]. We have shown the lack of methodological quality in most existing studies aiming at creating fetal brain charts. Most studies have significant risks of bias, leading to large differences in size charts of normal brain structures. This provides the controversial background context to studies that suggest that differences in fetal brain measurements exist due to, for example, maternal ethnicity, country of origin, or fetal sex: it is not possible to ascribe such differences to biological causes when basic methods of study design, statistical analyses, and reporting are not optimised and when different studies utilise widely different methods.

This review of the literature has shown that 40 out of 42 studies had a low risk of bias in no more than 60% of the quality criteria. In order to allow a unified approach to clinical practice and research, we suggest using charts that have an optimal study design, use a prescriptive approach, and a methodology that is at low risk of bias, including fetuses from low-risk populations worldwide, and that follow infants up for developmental assessment to confirm that the population was appropriate for the construction of these charts.

## Figures and Tables

**Table 1:** Main characteristics of studies included.

Study	Year	Country	Structure	Plane	Calipers Methods	TA or Tva	2D or 3D Acquisition	Study Design	Data Collection	Observation Period (Months)	Sample Size	GA Range (Weeks)	Quality Score
Alagappan [7]	1994	USA	PV	TV	NA	NA	2D	NA	R	NA	500	11–40	2
Almog [8]	2003	Israel	PV	TV	inn to inn	TA	2D	CS	P	NA	427	20–40	14
Alonso [9]	2010	Spain	POF SF	NA TV	NA NA	TA	2D	NA	P	NA	180	19–30	10
Alves [10]	2013	Brazil	POF SF	TV TV	out to inn† inn to inn	TA	3D	CS	P	17	393	22–33	16
Araujo Jùnion [11]	2014	Brazil	CM	TC	inn to inn	TA	2D	CS	R	83	3862	18–24	14
Araujo Jùnion [12]	2015	Brazil	TCD	TC	out to out	TA	2D	CS	R	84	3772	18–24	14
Brown [13]	2013	Canada	CM	TC	NA	TA	2D	CS	R	24	4750	15–32	14
Cardoza [14]	1988	USA	PV	TV	NA	TA	2D	NA	R	NA	100	14–38	2
Chang [15]	2000	Taiwan	TCD	NA	NA	TA	3D	CS	P	NA	223	20–40	10
Chavez [16]	2003	USA	TCD	NA	out to out	NA	2D	CS	R	96	2010	14–38	12
Chen [17]	2017	China	POF SF	NA TV	on to inn on to inn	TA	2D	CS	P	12	746	18–41	7
Eze [18]	2017	Nigeria	TCD	TC	out to out	TA	2D	CS	P	10	697	14–40	8
Farrell [19]	1994	USA	PV	TV	on to on	NA	2D	NA	P	NA	662	12–40	3
Goel [20]	2010	India	TCD	TC	out to out	NA	2D	NA	P	NA	50	14–40	6
Goldstein [21]	1987	USA/Italy	TCD	TC	out to out	NA	2D	CS	P	NA	335	13–40	8
Goldstein [22]	1988	USA	AV	TV	inn to inn	NA	2D	CS	P	NA	179	15–40	9
Goldstein [23]	1990	USA	PV	TV	on to on	NA	2D	NA	P	NA	160	15–40	7
Hata [24]	1989	Japan	TCD	TC	NA	NA	2D	NA	P	NA	116	17–40	8
Hayata [25]	2015	Japan	TCD	TC	NA	TA	2D	NA	P	8	150	14–36	9
Hilpert [26]	1995	USA	PV	NA	on to on	NA	2D	NA	P	6	537	13–42	2
Ishola [27]	2016	Nigeria	PV	TV	NA	TA	2D	CS	P	12	400	14–40	12
Joshi [28]	2010	Nepal	TCD	NA	out to out	NA	2D	CS	P	12	594	15–38	10
Köktener [29]	2007	Turkey	CM	NA	inn to inn	TA	2D	CS	P	NA	194	16–24	9
Köktener [30]	2012	Turkey	PV	TV	NA	TA	2D	NA	P	NA	338	15–25	7
Koning [31]	2017	Netherlands	TCD	NA	NA	TA and Tva	3D	L	P	17	166	9–32	16
Lei [32]	1998	China	PV TCD	NA TC	NA out to out	NA	2D	CS	P	24	5496	16–40	8
Mahony [33]	1984	USA	CM	TC	inn to inn	NA	2D	CS	P	1	155	15–36	4
Medvedev [34]	2017	Russia	PV TCD CM	TV TC TC	inn to inn out to out inn to inn	TA	3D	NA	R	NA	385	16–28	3
Mittal [35]	2007	USA	SF	NA	on to inn	TA	3D	CS	R	54	200	12–41	12
Napolitano [36]	2020	Brazil, India, Kenya, Italy, UK	POF SF AV PV CM	TT TT TV TV TC	inn to inn inn to inn inn to inn inn to inn inn to inn	TA	3D	L	P	84	442	14–42	27
Passos [37]	2015	Brazil	CM	TC	inn to inn	NA	3D	CS	P	17	224	17–29	15
Peixoto [38]	2016	Brazil	PV	TV	inn to inn	TA	2D	CS	R	36	512	16–41	15
Rodriguez-Sibaja [39]	2020	Brazil, India, Kenya, Italy, UK	TCD	TC	out to out	TA	3D	L	P	84	1130	14–42	25
Salomon [40]	2007	France	PV	TV	inn to inn	TA and Tva	2D	CS	P	49	4769	17–36	13
Serhatlioglu [41]	2003	Turkey	TCD CM	TC TC	NA inn to inn	TA	2D	CS	P	NA	130	16–38	8
Smith [42]	1986	UK	TCD CM	TC TC	NA inn to inn	NA	2D	CS	P	NA	107	14–32	12
Snijders [43]	1994	UK	AV PV TCD CM	TV TV TC TC	NA NA NA NA	NA	2D	CS	R	72	1040	14–40	12
Spinelli [44]	2019	Italy, Switzerland	SF	TT	inn to inn	TA	2D	CS	P	NA	329	18–33	16
Takano [45]	2018	Japan	TCD	TC	out to out	NA	2D	CS	R	60	340	14–40	10
Uerpairojkit [46]	2001	Thailand	TCD	TC	out to out	NA	2D	L	P	12	153	14–40	8
Verburg [47]	2008	Netherlands	TCD	TC	out to out	TA	2D	L	P	46	8313	16–36	17
Vinkesteijn [48]	2000	Netherlands	TCD	TC	out to out	NA	2D	CS	R	24	360	17–34	10

† outer margin of the fissure to internal margin of the calvarium. TA = transabdominal; Tva = transvaginal; POF = parieto-occipital fissure; SF = Sylvian fissure; AV = anterior ventricle; PV = posterior ventricle; TCD transcerebellar diameter; CM cisterna magna; TT = transthalamic; TV = transventricular; TC = transcerebellar; NA = not available; inn = inner; out = outer; CS = cross sectional; L = longitudinal; P = prospective; R = retrospective.

**Table 2:** Comparison of centiles value.

			20 Weeks				28 Weeks				32 Weeks			
			Centile			SD	Centile			SD	Centile			SD
Study	GA Included	Score	5	50	95		5	50	95		5	50	95	
**POF studies**														
Napolitano [36] 2020	14–42	27	2.7	4.2	5.8	0.95	4.1	6.1	8.2	1.27	4.6	6.8	9.0	1.34
Alves [10] 2013	22–36	16					15	16.7	18.3	1.65	17.8	19.4	21.1	1.65
Alonso [9] 2010	19–30	10		1.8	4.0	1.35	6.6	8.8	11.1	1.35				
**SF studies**														
Napolitano [36] 2020	14–42	27	3.5	5.9	8.2	1.42	8.0	11	14.1	1.85	9.9	13.1	16.3	1.96
Alves [10] 2013	22–36	16					8.0	10.4	12.8	1.65	9.5	12.3	15.0	1.65
Spinelli [44] 2019	18–33	16	5.7	6.9	8.1	0.7	10.3	12.1	13.9	1.1	11.4	13.5	15.6	1.3
Mittal [35] 2007	12–41	12	4.9	5.5	6.1		8.2	8.8	9.5		9.8	10.5	11.2	
Alonso [9] 2010	19–30	10	4.7	6.6	8.5	1.15	10.5	12.4	14.3	1.15				
**AV studies**														
Napolitano [36] 2020	14–42	27	4.9	6.9	8.9	1.2	5.8	7.8	9.8	1.2	6.5	8.4	10.4	1.2
Snijders [43] 1994	14–40	12	5.9	7.4	8.9	0.90	7.0	8.4	9.9	0.90	7.5	9.0	10.4	0.90
Goldstein [22] 1988	15–40	9	6.6	8.2	9.9		7.8	9.5	11.1		8.8	10.5	12.1	
**PV studies**														
Napolitano [36] 2020	14–42	27	4.1	6.3	8.5	1.36	3.1	5.8	8.5	1.63	2.7	5.7	8.6	1.77
Peixoto [38] 2016	16–41	15	3.7	5.5	7.3		2.9	5.1	7.3		2.5	4.9	7.3	
Almog [8] 2003	20–40	14	4.8	5.9	8.5	1.12	4.4	6.4	9.1	1.26	4.2	6.8	8.9	1.46
Salomon [40] 2007	17–36	13		6.8	8.8	0.15		6.1	8.8	0.22		6.5	9.1	0.21
Ishola [27] 2016	14–40	12	5.2	6.2	7.4		5.5	6.7	8.0		5.7	6.9	8.3	
Snijders [43] 1994	14–40	12	5.6	7.2	8.9	1.02	6.2	7.9	9.6	1.02	6.6	8.3	9.9	1.02
Goldstein [23] 1990	15–40	7	4.9	6.5	8.2		5.6	7.2	8.9		6.2	7.8	9.4	
Köktener [30] 2012	15–25	7	5.1	6.7	8.4									
**TCD studies**														
Rodriguez-Sibaja [39] 2020	14–42	25	19	20.5	21.9	0.87	30.3	32.9	35.5	1.57	37.3	40.8	44.2	2.11
Verburg [47] 2008	16–36	17	18.6	20.3	22.0	1.03	30.3	33.0	35.7	1.64	37.8	41.0	44.2	1.95
Koning [31] 2017	9–32	16	19.1	20.8	22.4		32.9	34.6	36.2		40.8	42.4	44.1	
Araujo Jùnior [12] 2015	18–24	14	17.9	19.9	22.8									
Chavez [16] 2003	14–38	12	17.7	20.4	23.0	0.20	28.2	32.4	36.6	0.35	34.4	39.5	44.7	0.46
Smith [42] 1986	14–32	12	18.8	20.5	22.1		29.4	31.0	32.6		34.6	36.3	37.9	
Snijders [43] 1994	14–40	12	19.0	21.0	24.0	0.03	29.0	32.0	36.0	0.03	34.0	37.0	42.0	0.03
Chang [15] 2000	20–40	10	15.4	20.3	25.3		27.8	32.7	37.7		34.0	38.9	43.8	
Joshi [28] 2010	15–38	10	19.1	20.7	22.3		31.3	32.9	34.6		37.4	39.1	40.7	
Takano [45] 2018	14–40	10	17.9	19	20	0.6	28.6	31.2	33.7	1.6	34	37.3	40.6	2
Vinkesteijn [48] 2000	17–34	10	18.8	20.7	22.8		29.3	32.3	35.7		36.7	40.4	44.6	
Hayata [25] 2015	14–36	9	19.3	19.9	20.5	0.36	29.4	31.7	34.0	1.40	35.8	38.1	40.4	1.40
Goldstein [21] 1987	13–40	8		20.0				31.0				38.0		
Hata [24] 1989	17–40	8	19.9	21.5	23.2		31.4	33.0	34.7		37.2	38.8	40.4	
Lei [32] 1998	16–40	8	19.0	21.0	25.8		25.8	36.6	34.5		32.0	43.0	44.9	
Serhatlioglu [41] 2003	16–38	8	20.0	21.7	23.3		32.9	34.6	36.2		39.7	41.3	43.0	
**CM studies**														
Napolitano [36] 2020	14–42	27	3.0	4.5	6.8	1.39	4.0	6.0	8.9	1.76	4.3	6.4	9.5	1.87
Passos [37] 2015	17–29	15	4.3	6.0	8.0		5.6	8.0	11.1					
Araujo Jùnior [11] 2014	18–24	14	2.9	4.7	6.5									
Brown [13] 2013	15–32	14	4.3	5.9	7.9	1.23	6.3	7.9	9.9	1.23	7.0	8.7	10.3	1.23
Snijders [43] 1994	14–40	12	3.3	5.1	7.2	0.04	4.7	6.8	9.1	0.04	5.2	7.3	9.7	0.04
Smith [42] 1986	14–32	12	4.2	5.8	7.5		6.7	8.3	10.0		7.9	9.6	11.2	
Köktener [29] 2007	16–24	9	2.7	4.3	6.0									
Serhatlioglu [41] 2003	16–38	8	3.0	4.7	6.3		4.7	6.3	7.9		4.9	6.6	8.2	

Centiles highlighted in white are reported in the relative study; centiles highlighted in grey are calculated. Studies are reported in descending quality score. SD= standard deviation; POF = parieto-occipital fissure; SF = Sylvian fissure; AV = anterior ventricle; PV = posterior ventricle; TCD = transcerebellar diameter; CM = cisterna magna; GA = gestational age.

## Supplementary Materials

The following are available online at https://www.mdpi.com/article/10.3390/diagnostics11060916/s1, Table S1: Search strategy, Table S2: List of methodological quality criteria, Table S3: Excluded studies after full paper review, Table S4: Risk of bias score.
