# Supplementary material for: A Systematic Review of Methodology Used in Studies Aimed at Creating Charts of Fetal Brain Structures
Source: Diagnostics (Basel). 2021 May 21;11(6):916. doi: 10.3390/diagnostics11060916 (PMC8223776; doi:10.3390/diagnostics11060916)
Supplement: Supplementary file 1 [file diagnostics-11-00916-s001.zip › diagnostics-1195319-supplementary.pdf]

diagnostics

MDPI

Table S1: Search strategy.

The search included a combination of title/abstract keywords and subject headings describing the following concepts – prenatal ultrasound, brain structure, fetal growth and reference standards. We didn't apply any language or publication restrictions. The full strategy for Medline was adapted for the other databases and these are available on request. In addition, we looked at references lists of included studies and relevant reviews.

# ▲	Searches
1	Ultrasonography, Prenatal/
2	(ultrasound or ultrasonogra† or ultra-sound or ultra-sonogra† or sonogra† or echograph† or echo-gram?).mp.
3	ultrasonography.fs.
4	2 or 3
5	(fetal or foetal or fetus† or foetus or prenatal† or pre-natal†).mp.
6	4 and 5
7	1 or 6
8	†brain/ or cerebral ventricles/ or lateral ventricles/ or cisterna magna/ or cranial fossa, posterior/ or exp cerebellum/
9	(brain? or cerebell† or transcerebell† or cerebral).ti.
10	(cerebellar or transcerebellar or cerebellum or cerebral cortex).ti,ab.
11	(posterior fossa or cisterna magna).ti,ab.
12	(sylvian fissure or lateral sulcus or lateral fissure or perisylvian cortex or cereb† fissure? or brain fissure? or parietooccipital fissure? or parieto-occipital fissure? or parietooccipital sulcus or parieto-occipital sulcus).ti,ab.
13	(lateral adj2 ventric†).ti,ab.
14	(brain or cereb† or lateral).mp. and ((anterior or posterior) adj2 ventric†).ti,ab.
15	((anterior or posterior) adj2 horn?).ti,ab.
16	8 or 9 or 10 or 11 or 12 or 13 or 14 or 15
17	"Embryonic and Fetal Development"/ or fetal development/ or gestational age/
18	Fetal Growth Retardation/
19	gestational age.ti,ab.
20	((fetal or foetal or fetus† or foetus or embryo†) adj2 growth).ti,ab.
21	((fetal or foetal or fetus† or foetus or embryo†) and (growth adj2 (normal or restrict† or retard†))).ti,ab.
22	17 or 18 or 19 or 20 or 21
23	reference standards/ or reference values/
24	"reproducibility of results"/
25	"Predictive Value of Tests"/
26	observer variation/
27	((reference or normal) adj2 (range? or standard? or value?)).ti,ab.
28	((reference or growth) adj2 (chart? or curve?)).ti,ab.
29	(correlat† or reproducib† or variation? or validat†).ti,ab.
30	(nomogram? or nomograph?).ti,ab.
31	(biometry or biometric?).ti,ab.

32	(percentile? or centile?).ti,ab.
33	23 or 24 or 25 or 26 or 27 or 28 or 29 or 30 or 31 or 32
34	7 and 16 and 22 and 33
35	((cerebellar or transcerebellar or cerebellum) adj2 diameter).ti,ab.
36	((posterior fossa or cisterna magna) adj2 diameter).ti,ab.
37	35 or 36
38	35 and 22
39	34 or 38

† indicates keyword truncation.

Table S2: List of methodological quality criteria.

	**Low risk of bias**	**High risk of bias**
**1. STUDY DESIGN**		
1.1. Design	Clearly described as either cross-sectional or longitudinal	Mixture of cross-sectional and longitudinal data; or not reported
1.2 Sample selection	Population based study where there are attempts to identify and clearly define populations from a specific geographic area; from this underlying population, women are selected either consecutively or at random	Not population based; convenience sampling; arbitrary recruitment; or not reported
1.3 Number of occasions each fetus was measured (only for cross-sectional studies)	Each fetus was measured and included only once	Some fetuses were measured and included more than once
1.4. Method of selecting the gestational ages at which the fetuses were measured and reason(s) for choosing a particular number of serial measurements (only for longitudinal studies)	Interval of measures prospectively pre-specified and justified<br>Clear documentation of the intended number of serial measurements	Interval of measures not prospectively pre-specified and justified or not reported<br>No clear documentation of the intended number of serial measurements
1.5 Inclusion /Exclusion criteria	*The study made it clear that women at high risk of pregnancy complications were not included; and that women with abnormal outcome were excluded, i.e. an effort was made to include "normal" outcome as best possible*<br>As a minimum the study population should exclude:<br>- multiple pregnancy<br>- fetuses with congenital structural or chromosomal anomalies<br>- fetal death<br>- women with disorders that may affect fetal growth and pregnancy outcome (at least should specify exclusion of women with pre-existing hypertension, diabetes mellitus, renal disease, smoking, BMI>35)<br>- pregnancy complications (at least preeclampsia)	*The study population included both low-risk and high-risk pregnancies or women with abnormal outcome were not excluded*<br>Study population that did not exclude fetuses or women with the characteristics previously described.<br>Exclusions which would have a direct effect on the estimated percentiles, such as fetuses found at birth to be large or small for dates
1.6. Neonatal and infant outcome	As a minimum the description should include:	*Neonatal and infant outcome not reported*

	Low risk of bias	High risk of bias
	- birth weight, length, head circumference at birth<br>- NICU admission and neonatal mortality<br>- long term development at 1 year of age	
1.7. Sample size	A priori determination / calculation of sample size and justification	Lack of a priori sample size determination / calculation and justification
1.8. Data collection	Prospective study and ultrasound data collected specifically for the purpose of constructing charts of brain structures	Retrospective study, or data not collected specifically for the purpose of constructing charts, or unclear (e.g. use of routinely collected data)
1.9. Pregnancy dating	Method clearly described: known LMP and regular menstrual cycles AND a sonogram before 14 weeks demonstrating a crown-rump length (CRL) that corroborates LMP dates	Not described clearly<br>GA assessment at >14 weeks; or GA assessment not including USS verification
1.10. Collection of data on gestational age at inclusion	The gestational age was calculated precisely to the day	Truncation of gestational age to the number of "completed weeks"
**2. STATISTICAL METHODS**		
2.1. Number of measurements taken for each biometric variable	More than one measurement per fetus per scan	Single measure or not specified
2.2. Description of statistical methods	Clearly described and identified	Not clearly described and identified
2.3. Assessment of increasing variability of the data with gestation	Performed	Not performed
2.4. Assessment of goodness of fit of the models	A test of goodness-of-fit of the models was reported	Goodness-of-fit of models was not reported
2.5. Scatter diagram of the data	Study included scatter diagrams of the data	Study did not include scatter diagrams of the data
2.6. Change of reference centiles across GA	Smooth change	Not smooth change
2.7. Scatter diagram of the data with the fitted centiles superimposed	Study included scatter diagrams of the data with the percentiles superimposed	Study did not include scatter diagrams of the data with the percentiles superimposed
2.8. Methods used to estimate age-specific reference intervals for brain measurements	Estimated "Mean and SD model", smoothed crude percentiles, or "LMS method" per week	Inadequate
**3. REPORTING METHODS**		

	Low risk of bias	High risk of bias
3.1. Characteristics of study population	Presented in a table or clearly described and includes minimum dataset of age, weight, height or BMI and parity	Not presented in a table or not clearly described, or does not contain minimum data set
3.2 Description of number approached / enrolled	Described	Not described
3.3. Ultrasound machine(s) used	Clearly specified	Not clearly specified
3.4 Probe Type (Transvaginal or Transabdominal)	Reported	Not reported
3.5. Multiple sonographers that took the measurements	Reported	Unreported or single sonographer
3.6. Description of measurement techniques	The study described sufficient and unambiguous details of the measurement techniques used for fetal size parameters, including imaging plane and calliper application method	The study did not describe sufficient and unambiguous details of the measurement techniques used for fetal size parameters
3.7 Measurements acquired blindly	Sonographer was blind to measurements	Unreported or sonographer was not blind to measurements
3.8. Standardization of the sonographers prior to the study	Performed	Not performed
3.9. Contains quality control measures	Should include the following<br>- Assessment of intra- observer and inter – observer variability using Bland-Altman plots<br>- Image review and storing	Does not contain quality control measures
3.10. Report of observed mean and SD of each measurement and the sample size for each week of gestation	Presented in a table or clearly described	Not presented in a table or not clearly described
3.11. Report of regression equations for the mean (and SD if relevant) for each measurement	Reported	Not reported

NICU= neonatal intensive care unit; LMP= last mestrual period; CRL crown-rump length; GA= gestational age; USS= ultrasound; SD= standard deviation.

Table S3: Excluded studies after full paper review.

**Author**	**Reasons for exclusion**
Albers 2018	TCD measurement with no aim of construction of growth chart
Bansal 2014	TCD measurement for prediction of GA
Chinn 1983	PV abnormal appearance
Cohen-Sacher 2006	Overview on brain sulcation
Denkhaus 1979	SF and AV measured in a different way and function of BPD
Filly 1994	Comment on another paper on PV
Haddad 2001	PV measurement with no aim of construction of growth chart
Hadlock 1981	Anatomy of the lateral ventricle
Heiserman 1991	PV measurement with no aim of construction of growth chart
Hill 1990	TCD measurement in large for GA fetuses
Hill 1990	TCD measurement in small for GA fetuses
Jacquemyn 2000	TCD measurement with no aim of construction of growth chart
Jeanty 1981	Lateral ventricle and hemispheric width ratio
Johnson 1980	Lateral ventricle and hemispheric width ratio
Jörgensen 1986	Lateral ventricle width
Koothan 2018	TCD measurement with no aim of construction of growth chart
Lustig-Gillman 1984	AV measured in a different way
McGahan 1983	Ventricle measurements other than AV and PV
McLeary 1984	TCD measurement with no aim of construction of growth chart
Meyer 1993	TCD and abdominal circumference ratio
Monteagudo 1993	PV measured on a plane other than transverse
Monteagudo 1997	Overview on brain sulcation
Nardozza 2014	CM volume growth chart
Patel 1995	PV measurement with no aim of construction of growth chart
Pilu 1989	PV measurement with no aim of construction of growth chart
Pistorius 2010	Overview on brain sulcation
Pretorius 1986	Lateral ventricle width
Siedler 1987	PV and SF measurement with no aim of construction of growth chart
Tao 2008	CM measurement with no aim of construction of growth chart
Zador 1988	Overview on fetal anatomy
Reddy 2017	TCD measurement for prediction of GA

TCD= transcerebellar diameter; GA=gestational age; PV= posterior ventricle; SF= Sylvian fissure; AV= anterior ventricle; BPD = biparietal diameter; CM= cisterna magna.

Table S4: Risk of bias score. 0= high risk of bias; 1= low risk of bias

Domain: Study design	1.1 Design	1.2 Sample	1.3 No multiple scans	1.4 Scans interval	1.5 Exclusion criteria	1.6 Neonatal and infant	1.7 Sample size	1.8 Data collection	1.9 Pregnancy dating	1.10 Gestational age calculation	Total
Alagappan, 1994	0	0	0	0	0	0	0	0	0	0	0
Almog, 2003	1	0	1	0	0	0	0	1	1	0	4
Alonso, 2010	0	0	0	0	0	0	0	1	0	1	2
Alves, 2013	1	0	1	0	0	0	0	1	0	1	4
Araujo Jùnior, 2014	1	0	1	0	0	0	0	0	0	1	3
Araujo Jùnior, 2015	1	0	1	0	0	0	0	0	0	1	3
Brown, 2013	1	0	1	0	0	0	0	0	0	1	3
Cardoza, 1988	0	0	0	0	0	0	0	0	0	0	0
Chang, 2000	1	0	1	0	0	0	0	1	0	0	3
Chavez, 2003	1	0	1	0	0	0	0	0	0	0	2
Chen, 2017	1	0	1	0	0	0	0	1	0	0	3
Eze, 2017	1	0	0	0	0	0	1	1	0	0	3
Farrell, 1994	0	0	0	0	0	0	0	1	0	0	1
Goel, 2010	0	0	0	0	0	0	0	1	0	1	2
Goldstein, 1987	1	0	1	0	0	0	0	1	0	1	4
Goldstein, 1988	1	0	1	0	0	0	0	1	0	0	3
Goldstein, 1990	0	0	0	0	0	0	0	1	0	1	2
Hata, 1989	0	0	0	0	0	0	0	1	0	1	2
Hayata, 2015	0	0	0	0	0	0	0	1	0	1	2
Hilpert, 1995	0	0	0	0	0	0	0	1	0	0	1
Ishola, 2016	1	0	1	0	0	0	0	1	0	0	3
Joshi, 2010	1	0	1	0	0	0	0	1	0	0	3
Köktener, 2007	1	0	0	0	0	0	0	1	1	1	4
Köktener, 2012	0	0	0	0	0	0	0	1	0	1	2
Koning, 2018	1	0	0	0	0	0	0	1	1	1	4
Lei, 1998	1	0	1	0	0	0	0	1	0	0	3
Mahony, 1984	1	0	1	0	0	0	0	1	0	0	3
Medvedev, 2017	0	0	0	0	0	0	0	0	0	1	1
Mittal, 2007	1	0	0	0	0	0	0	0	0	0	1
Napolitano, 2020	1	1	1	1	1	1	1	1	1	1	10
Passos, 2015	1	0	1	0	0	0	0	1	1	1	5
Peixoto, 2016	1	0	1	0	0	0	0	0	1	1	4
Rodriguez-Sibaja, 2020	1	1	1	1	1	1	1	1	1	1	10
Salomon, 2007	1	0	1	0	0	0	0	1	0	0	3
Serhatlioglu, 2003	1	0	1	0	0	0	0	1	0	0	3
Smith, 1986	1	0	1	0	0	0	0	1	0	1	4
Snijders, 1994	1	0	0	0	0	0	0	0	0	1	2
Spinelli, 2019	1	0	0	0	0	0	1	1	1	0	4
Takano, 2018	1	0	1	0	0	0	0	0	0	0	2
Uerpairojkit, 2001	1	0	0	1	0	0	0	1	0	0	3
Verburg, 2008	1	1	0	1	0	0	0	1	0	1	5
Vinkesteijn, 2000	1	0	1	0	0	0	0	0	0	0	2

Domain: Statistical methods / Study	2.1 Number of measurement	2.2 Description of statistical	2.3 Increasing variability	2.4 Goodness of fit	2.5 Scatter diagram of the data	2.6 Change of centiles	2.7 Scattered diagram with superimposed	2.8 Methods to estimate reference	Total
Alagappan, 1994	0	0	1	0	0	0	0	0	1
Almog, 2003	1	1	0	0	1	1	1	1	6
Alonso, 2010	0	1	0	0	1	1	1	0	4
Alves, 2013	0	1	1	1	1	1	1	1	7
Araujo Jùnior, 2014	0	1	1	0	1	1	1	1	6
Araujo Jùnior, 2015	0	1	1	0	1	1	1	1	6
Brown, 2013	0	1	1	1	1	1	1	1	7
Cardoza, 1988	0	0	0	0	0	0	0	0	0
Chang, 2000	0	1	0	1	1	0	0	1	4
Chavez, 2003	0	1	1	1	1	1	1	1	7
Chen, 2017	0	0	0	0	0	0	0	0	0
Eze, 2017	1	0	0	0	0	0	0	0	1
Farrell, 1994	0	0	0	0	0	0	0	0	0
Goel, 2010	0	0	0	0	1	0	0	0	1
Goldstein, 1987	0	0	0	0	1	0	0	1	2
Goldstein, 1988	0	1	0	0	1	0	0	1	3
Goldstein, 1990	0	0	0	0	1	0	0	1	2
Hata, 1989	0	0	1	0	1	1	1	0	4
Hayata, 2015	0	0	0	0	1	1	1	1	4
Hilpert, 1995	0	0	0	0	0	0	0	0	0
Ishola, 2016	0	1	0	0	1	1	1	1	5
Joshi, 2010	1	1	1	0	0	0	0	1	4
Köktener, 2007	0	0	0	0	1	0	0	0	1
Köktener, 2012	0	0	0	0	1	0	0	0	1
Koning, 2018	1	1	1	1	1	1	1	1	8
Lei, 1998	0	0	0	0	0	0	0	1	1
Mahony, 1984	0	0	0	0	0	0	0	0	0
Medvedev, 2017	0	0	0	0	0	0	0	0	0
Mittal, 2007	1	1	0	1	1	1	1	1	7
Napolitano, 2020	0	1	1	1	1	1	1	1	7
Passos, 2015	0	1	1	0	1	1	1	1	6
Peixoto, 2016	0	1	1	0	1	1	1	1	6
Rodriguez-Sibaja, 2020	0	1	1	1	1	1	1	1	7
Salomon, 2007	0	1	1	1	0	1	0	1	5
Serhatlioglu, 2003	1	1	0	0	0	0	0	0	2
Smith, 1986	0	1	1	1	0	1	0	1	5
Snijders, 1994	0	1	1	1	1	1	1	1	7
Spinelli, 2019	0	1	1	1	1	1	1	1	7
Takano, 2018	0	1	1	1	0	1	0	1	5
Uerpairojkit, 2001	0	0	1	0	0	0	0	1	2
Verburg, 2008	0	1	1	1	1	1	1	1	7
Vinkesteijn, 2000	0	1	1	0	1	1	1	1	6

Domain: Reporting methods / Study	3.1 Characteristics	3.2 Number approached and enrolled	3.3 Ultrasound equipment	3.4 Probe type	3.5 Multiple sonographers reported	3.6 Description of measurement techniques	3.7 Blind measurement	3.8 Standardised sonographers	3.9 Quality control	3.10 Observed value	3.11 Equation reported	Total
Alagappan, 1994	0	0	0	0	1	0	0	0	0	0	0	1
Almog, 2003	0	0	1	1	0	1	0	0	0	0	1	4
Alonso, 2010	0	0	1	1	0	0	0	0	1	0	1	4
Alves, 2013	0	0	1	1	0	1	0	0	1	0	1	5
Araujo Jùnior, 2014	0	0	0	1	1	1	0	0	0	1	1	5
Araujo Jùnior, 2015	0	0	0	1	1	1	0	0	0	1	1	5
Brown, 2013	0	0	1	1	1	0	0	0	0	0	1	4
Cardoza, 1988	0	0	0	1	1	0	0	0	0	0	0	2
Chang, 2000	0	0	1	0	1	0	0	0	0	0	1	3
Chavez, 2003	0	0	1	0	0	0	0	0	0	1	1	3
Chen, 2017	0	0	1	1	0	0	0	0	1	1	0	4
Eze, 2017	0	0	1	1	0	1	0	0	0	1	0	4
Farrell, 1994	0	0	1	0	0	1	0	0	0	0	0	2
Goel, 2010	0	0	1	0	0	1	0	0	0	0	1	3
Goldstein, 1987	0	0	1	0	0	1	0	0	0	0	0	2
Goldstein, 1988	0	0	1	0	0	1	0	0	0	0	1	3
Goldstein, 1990	0	0	1	0	0	1	0	0	0	0	1	3
Hata, 1989	0	0	1	0	0	0	0	0	0	0	1	2
Hayata, 2015	0	0	1	1	0	0	0	0	0	0	1	3
Hilpert, 1995	0	0	0	0	1	0	0	0	0	0	0	2
Ishola, 2016	0	0	1	1	0	0	0	0	0	1	1	4
Joshi, 2010	0	0	1	0	0	0	0	0	0	1	1	3
Köktener, 2007	0	0	1	1	0	0	0	0	0	1	1	4
Köktener, 2012	0	0	1	1	0	0	0	0	0	1	1	4
Koning, 2018	1	0	1	1	0	0	0	0	0	0	1	4
Lei, 1998	0	0	1	0	1	0	0	1	0	1	0	4
Mahony, 1984	0	0	0	0	0	1	0	0	0	0	0	1
Medvedev, 2017	0	0	1	0	0	1	0	0	0	0	0	2
Mittal, 2007	0	0	1	1	0	0	0	0	1	0	1	4
Napolitano, 2020	1	1	1	1	1	1	1	1	1	0	1	10
Passos, 2015	0	0	1	0	0	1	0	0	1	0	1	4
Peixoto, 2016	1	0	1	0	1	1	0	0	0	0	1	5
Rodriguez-Sibaja, 2020	1	1	1	1	0	1	1	1	0	0	1	8
Salomon, 2007	0	0	1	1	0	1	0	0	0	1	1	5
Serhatlioglu, 2003	0	0	1	1	0	0	0	0	0	0	1	3
Smith, 1986	0	1	1	0	0	0	0	0	0	0	1	3
Snijders, 1994	0	0	1	0	1	0	0	0	0	0	1	3
Spinelli, 2019	1	0	1	1	0	1	0	0	0	0	1	5
Takano, 2018	0	0	1	0	0	1	0	0	0	0	1	3
Uerpairojkit, 2001	0	0	1	0	0	1	0	0	0	1	0	3
Verburg, 2008	0	0	1	0	1	1	0	1	0	0	1	5
Vinkesteijn, 2000	0	0	1	0	0	1	0	0	0	0	0	2
